# Polypharmacy in psychiatry and weight gain: longitudinal study of 832 patients hospitalized for depression or schizophrenia, along with data of 3180 students from Europe, the U.S., South America, and China

**DOI:** 10.1007/s00406-024-01767-2

**Published:** 2024-03-10

**Authors:** H. H. Stassen, S. Bachmann, R. Bridler, K. Cattapan, E. Seifritz

**Affiliations:** 1 Department of Psychiatry, Psychotherapy and Psychosomatics, Institute for Response-Genetics, Psychiatric University Hospital, 8032 Zurich, Switzerland; 2 Department of Psychiatry, Psychotherapy, and Psychosomatics, University of Halle, 06112 Halle, Germany; 3 Germany and Clienia AG, Psychiatric Hospital, 9573 Littenheid, Switzerland; 4 Department of Psychiatry, Geneva University Hospitals, 1226 Thônex, Switzerland; 5 Sanatorium Kilchberg, 8802 Kilchberg, Switzerland; 6 University Hospital of Psychiatry and Psychotherapy, University of Bern, Bern, Switzerland; 7 Department of Psychiatry, Psychotherapy and Psychosomatics, Psychiatric University Hospital, 8032 Zurich, Switzerland

**Keywords:** Polypharmacy, Monotherapy, Antidepressants, Antipsychotics, Efficacy, Side effect profiles, Concurrent medications, Weight gain

## Abstract

Epidemiologic data indicate that overweight and obesity are on the rise worldwide. Psychiatric patients are particularly vulnerable in this respect as they have an increased prevalence of overweight and obesity, and often experience rapid, highly undesirable weight gain under psychotropic drug treatment. Current treatment strategies in psychiatry are oriented towards polypharmacy, so that the information on drug-induced weight gain from earlier monotherapy studies is of very limited validity. We have analyzed the longitudinal data of 832 inpatients with ICD-10 diagnoses of either F2 (schizophrenia; n = 282) or F3 (major depression; n = 550) with the goal of ranking treatment regimens in terms of weight gain, side effects, and response to treatment. The patient data were complemented by the data of 3180 students aged 18–22 years, with which we aimed to identify factors that enable the early detection and prevention of obesity and mental health problems. After 3 weeks of treatment, 47.7% of F2 patients and 54.9% of F3 patients showed a weight gain of 2 kg and more. Major predictive factors were “starting weight” (r = 0.115), “concurrent medications” (r = 0.176), and “increased appetite”(r = 0.275). Between 11 and 30% of the observed variance in weight gain could be explained by these factors, complemented by sex and age. The comparison between monotherapy (n = 409) and polypharmacy (n = 399) revealed significant drawbacks for polypharmacy: higher weight gain (*p* = 0.0005), more severe side effects (*p* = 0.0011), and lower response rates (F2: *p* = 0.0008); F3: *p* = 0.0101). The data of 3180 students made it clear that overweight and obesity often begin early in life among those affected, and are interconnected with personality traits, while increasing the risk of developing psychosomatic disturbances, mental health problems, or somatic illnesses. Although the available data did not readily lead to a comprehensive, clinically applicable model of unwanted weight gain, our results have nevertheless demonstrated that there are ways to successfully counteract such weight gain at early stages of treatment.

## Background

Epidemiologic data indicate that overweight and obesity are on the rise worldwide, driven by modern lifestyles, unhealthy diets, alcohol consumption, lack of physical activity, and chronic stress, amongst others [1–3]. Along with bodily challenges in daily life, for example, when using public transportation, overweight and obesity have also led to a rise in related health complications, including diabetes mellitus, insulin resistance, renal dysfunction, chronic low-grade inflammation, cancer, cardiovascular disease, neurodegenerative abnormalities, depression, cognitive decline, Alzheimer's disease (AD), and other dementias [4–8]. As obesity and overweight constitute a global threat to general health, the World Health Organization (WHO: a United Nations agency working to promote health) has developed a “Global Strategy on Diet, Physical Activity, and Health” that involves a series of concrete steps to be implemented by governmental institutions [9]. Despite promising initiatives, this has not yet been able to stop the steady global spread of overweight and obesity.

Psychiatric patients are particularly vulnerable in this respect: on the one hand, the prevalence of overweight and obesity is increased among psychiatric patients compared to the general population, and on the other hand, medications with psychotropic substances very often lead to a rapid, highly undesirable weight gain. And worst of all, there is no long-term cure for a substantial proportion of patients, for example, for 50–60% of patients with schizophrenic disorders [10], and for 35–50% of patients with major depression [11, 12].

The weight gain induced by psychotropic drugs, such as antidepressants or antipsychotics, varies across substances as well as across subjects so that all available weight gain data are statistical in nature and do not necessarily apply to the individual case. A standard measure to counteract excessive weight gain under antidepressants, antipsychotics, or lithium, is therefore to switch a patient’s medication to substances that may cause less weight gain.

Current treatment strategies in psychiatry are oriented towards polypharmacy, that is, patients are no longer treated with one single medication but receive combinations of several antidepressants, antipsychotics, mood stabilizers, anxiolytics, hypnotics, antihistamines, and anticholinergics, along with other somatic treatments [13–21]. This has the consequence that any established information on drug-induced weight gain as derived from earlier monotherapy studies is of very limited validity or no longer applicable at all.

Given the elevated vulnerability of psychiatric patients regarding drug-induced weight gain, overweight and obesity, together with the immense psychological and somatic burdens resulting therefrom, this comprehensive project addressed the following questions through empirical data: (1) extent to which overweight and obesity are more prevalent among psychiatric patients compared to the normal population; (2) extent to which psychotropic drugs induce unwanted weight-gain; (3) differences in drug-induced weight gain between the diagnostic entities “schizophrenia” and “major depression”, as patients with schizophrenia receive antipsychotics as primary medication, and patients with major depression receive antidepressants as primary treatment; (4) differences between monotherapy and polypharmacy regarding drug-induced weight gain; (5) severity of side effects co-occurring with drug-induced weight gain; 6) factors that might predict drug-induced weight gain prior to the start of therapeutic intervention; (7) extent to which overweight and obesity start developing in early life and are socio-culturally independent; (8) factors that might influence overweight and obesity in early life. Answers to the above questions were expected to directly translate into clinical practice.

## Methods

### Patient sample I

In an earlier prospective study (“*Response-Genetics Wave-I*”: 2002–2010), we recruited 512 patients hospitalized at three residential mental health treatment centers with an ICD-10 diagnosis of either schizophrenic (“F2x.x”; n = 188; “F2 patients”) or depressive disorders (“F32.x/F33.x”; n = 324; “F3 patients”).^1^ This was a “naturalistic” observational study of psychiatric inpatients, designed to provide an accurate picture of actual treatment practices. In form of an add-on to clinical routine, the study aimed to assess today's acute inpatient treatment regimens regarding therapeutic strategies, medications, adverse side effects, time course of recovery, and efficacy of treatments. By design, this observational study had no influence whatsoever on treatment modalities. All new admissions with a suspected primary ICD-10 diagnosis of “F2x.x” or “F32.x/F33.x” were contacted by the study administrator (senior psychiatrist) and invited to participate. As there were up to 3 reviewers per center it rarely ever occurred that a patient could not enroll in the study due to lack of interviewer availability. In these extremely rare cases, the FIFO rule was employed. As the study was an add-on to clinical routine, the patients typically entered the study shortly after starting treatment. Final diagnoses were decided by consensus of two senior psychiatrists. All patients signed a written “informed consent” after having been informed about the aims of the project and that they can stop their participation at any time without any disadvantages. Psychopathology was assessed by specifically trained psychiatrists and psychologists to improve inter-rater agreement.

The study protocol included (1) up to 8 repeated measurements over 5 weeks assessing the time course of improvement through the 17/21-item Hamilton Depression Scale HAM-D [22], or the 30-item Positive and Negative Syndrome Scale PANSS [23]; (2) the assessment of a global side effect score along with body weight; and (3) the collection of blood samples for serum extraction and DNA isolation. The repeated assessments regarding the course of improvement were carried out at weekly intervals plus 2 additional assessments at the 3rd and 10th day.

The HAM-D instrument assesses the severity of depressive disorders by means of a single scale, while the PANSS instrument assesses the severity of schizophrenic disorders in terms of positive, negative, and general psychopathology scales. A minimum baseline score of at least 15 on the HAM-D17 Scale (primary “F32.x/F33.x” diagnoses), or of at least 21 on the general psychopathology PANSS-G Scale (primary “F2x.x” diagnoses), was required at entry into study. The PANSS-G scale was chosen in order to prioritize illness-related disabilities in daily functioning over acute productive symptomatology and longer persisting negative symptoms. Patients who did not meet the minimum baseline score criterion were excluded from analysis.

### Patient sample II

In a recent prospective study (“*Response-Genetics Wave-II*”: 2012–2020), we recruited 320 patients hospitalized at three residential mental health treatment centers with an ICD-10 diagnosis of either schizophrenic (“F2x.x”; n = 94; “F2 patients”) or depressive disorders (“F32.x/F33.x”; n = 226; “F3 patients”). This was a “naturalistic” observational study of psychiatric inpatients, designed to provide an accurate picture of actual treatment practices. In form of an add-on to clinical routine, the study aimed to assess today's acute inpatient treatment regimens regarding therapeutic strategies, medications, adverse side effects, time course of recovery, and efficacy of treatments. By design, this observational study had no influence whatsoever on treatment modalities. All new admissions with a suspected primary ICD-10 diagnosis of “F2x.x” or “F32.x/F33.x” were contacted by the study administrator (senior psychiatrist) and invited to participate. As there were up to 3 reviewers per center it rarely ever occurred that a patient could not enroll in the study due to lack of interviewer availability. In these extremely rare cases, the FIFO rule was employed. As the study was an add-on to clinical routine, the patients typically entered the study shortly after starting treatment. Final diagnoses were decided by consensus of two senior psychiatrists. All patients signed a written “informed consent” after having been informed about the aims of the project and that they can stop their participation at any time without any disadvantages. Psychopathology was assessed by specifically trained psychiatrists and psychologists to improve inter-rater agreement.

The study protocol included (1) assessments of previous history and overall social functioning through the 63-item SADS Syndrome Check List SSCL-16 and 83-item SADS-Supplement SSCL-SUPP (lifetime versions) [24]; (2) up to 8 repeated measurements over 5 weeks assessing the time course of improvement through the 17/21-item Hamilton Depression Scale HAM-D or the 30-item Positive and Negative Syndrome Scale PANSS; (3) up to 8 repeated measurements over 5 weeks assessing medication and unwanted side effects through the 46-item Medication and Side Effects Inventory MEDIS [25]; and (4) the collection of blood samples for serum extraction and DNA isolation. The repeated assessments regarding the course of improvement were carried out at weekly intervals plus 2 additional assessments at the 3rd and 10th day.

The syndrome-oriented instrument SSCL-16 extends the ICD-10 definitions by replacing the yes–no dichotomy of diagnostic schemata by the dimensional quantities «schizophrenic thought disorders», «delusions», «hallucinations», «ego consciousness», «incongruent affect», «anergia», «depressive syndrome», «manic syndrome», and «suicide», while the SSCL-SUPP measures the patients’ overall level of functioning, personality traits, somatization, and consumption behavior. The MEDIS instrument details side-effect clusters in a quantitative way with respect to «sleep», «appetite», «sexuality», «gastro-intestinal», «cardiac-respiratory», «autonomic», «psychosomatic», «neurological», and «cardiovascular» disturbances.

A minimum baseline score of at least 15 on the HAM-D17 Scale (primary “F32.x/F33.x” diagnoses), or of at least 21 on the general psychopathology PANSS-G Scale (primary “F2x.x” diagnoses), was required at entry into study. The PANSS G scale was chosen in order to prioritize illness-related disabilities in daily functioning over acute productive symptomatology and longer persisting negative symptoms. Patients who did not meet the minimum baseline score criterion were excluded from analysis.

### Study of 3180 students from Europe, the U.S., South America, and China

We have recruited a total of 3180 students at 8 different universities by setting up information stands at central locations on the university campuses for 5 days, where students in the age range between 18 and 22 years could get information about the goals of the project and sign up to participate in the study (typically 2–3% of eligible students declined). Recruitment was carried out at the following universities: (1) Bristol/UK [n = 210: 64 males, 146 females]; (2) Milano/Italy [n = 420: 212 males, 208 females]; (3) Valencia/Spain [n = 400: 202 males, 198 females]; (4) Lausanne/ Switzerland [n = 405: 130 males, 275 females]; (5) Zurich/Switzerland [n = 406: 221 males, 185 females]; (6) Pasadena/USA [n = 407: 180 males, 227 females]; (7) Cipolletti/ Argentina [n = 500: 138 males, 362 females]; and (8) Hangzhou/China [n = 432: 222 males, 210 females] [26–28].

The students were asked to fill out the 28-item Coping Strategies Inventory “COPE”, and the 63-item Zurich Health Questionnaire “ZHQ” which assesses the factors “regular exercises”, “consumption behavior”, “impaired physical health”, “psychosomatic disturbances”, and “impaired mental health”. Both instruments COPE and ZHQ are available in 6 languages through the website “https://ifrg.ch/instruments.php”, are strictly anonymous and do not collect any personal data.

The COPE instrument assesses basic coping behavior under chronic stress which is summarized by two scales “Activity” (activity-passivity) and “Defeatism” (defeatism-resilience). In calibration studies, these two scales explained > 65% of the observed inter-individual variation inherent in the 28 COPE items (> 43% by “activity”, > 22% by “defeatism”) [26–28]. “Activity” is best described through items like “turning to work”, and “coming up with a strategy”, whereas “Defeatism” is characterized by behavior like “giving up”, “using alcohol”, or “refusing to believe that this has happened”. “Passivity” is understood as negative scoring on the activity scale, and “resilience” as negative scoring on the defeatism scale. The term “resilience” encompasses all those endogenous mechanisms that support and maintain health, thereby enabling patients to cope with stressful situations.

Specifically, we were interested in the relationship between overweight and obesity on the one hand, and mental health, physical health, regular exercises, and consumption behavior on the other. The goal was to identify factors that enable the early detection and prevention of mental health problems, as well as of unwanted weight gain, overweight, and obesity.

### Statistical analyses

We used the Statistical Analysis Software SAS/STAT 9.4 by SAS Institute Inc. for repeated measurement analyses (PROCs ANOVA, CORR(PEARSON/SPEARMAN), FREQ(CHISQ), GLM, NPAR1WAY, TTEST; Bonferroni corrections where necessary, specifically for the correlation analysis between personality traits on the one hand, and the factors “regular exercises”, “consumption behavior”, “impaired physical health”, “psychosomatic disturbances”, and “impaired mental health” on the other), and the SPSS 28 Statistics Package by IBM, along with PROC *HPNEURAL* from SAS Enterprise Miner 15.1, for Neural Nets analyses.

We have followed the guidelines of the World Health Organization (WHO), which has studied body weight in great detail in terms of its influence on human health, thereby relying on the Body Mass Index (BMI) as a risk indicator of disease. We adopted the WHO classification of BMI: (1) BMI < 18.5 kg as «underweight»; (2) 18.5 kg ≤ BMI ≤ 24.9 kg as «normal weight»; (3) 25.0 kg ≤ BMI ≤ 29.9 kg as «overweight» or «pre-obesity»; and (4) BMI ≥ 30 kg as «obesity».

The patients’ weight gain was determined after 3 weeks of treatment. Patients, who dropped out prematurely but had weight measurements after 2 weeks of treatment, were also included in the analysis by means of the standard LOCF method (Last Observation Carried Forward). We distinguished five categories of weight gain “WG”: (1) weight loss: WG < − 2 kg; (2) “no change”: − 2 kg ≤ WG < + 2 kg; (3) “mild” weight gain: 2 kg ≤ WG < 5 kg; (4) “moderate” weight gain: 5 kg ≤ WG < 7.5 kg; and (5) “severe” weight gain: WG ≥ 7.5 kg. These categories were derived through an analysis of 577 healthy subjects regarding “typical” fluctuations in weight assessments at 14-day intervals [29]. The observed fluctuations were in the range of ± 1.2 kg (two standard deviations). That value was then rounded up to the next whole number, and we defined changes of ≥ 2 kg as significant “weight gain”, or “weight loss”. In accordance with this empirical measure, patients characterized a weight gain of 2 kg as clearly noticeable, unpleasant, and quite irritating. The proposed categories worked well in practice, giving a good impression of what is going on in terms of body weight. By contrast, the use of relative weight gain would have meant that overweight people with a 5 kg gain still fall into the group of patients without significant weight gain, thus downplaying drug-induced weight gain in an inappropriate way.

The global side effect score “GS” was categorized in the following way: GS ≤ 10: “no”, 10 < GS ≤ 20: “mild”, 20 < GS ≤ 40: “moderate”, 40 < GS ≤ 60: “severe”, and 60 < GS: “very severe” side effects.

In line with our previous studies in this field (cf. [30, 31]), we used scale-based cutoff values for the definition of response to treatment. “Response” under depression therapies was defined by a sustained^2^ 50% HAM-D17 baseline score reduction, and under schizophrenia therapies by a sustained 40% PANSS-P baseline score reduction. Similarly, we defined “onset of improvement” by a sustained 20% HAM-D17, or a 20% PANSS-P baseline score reduction respectively.

### Neural nets

**Fig. 1:**
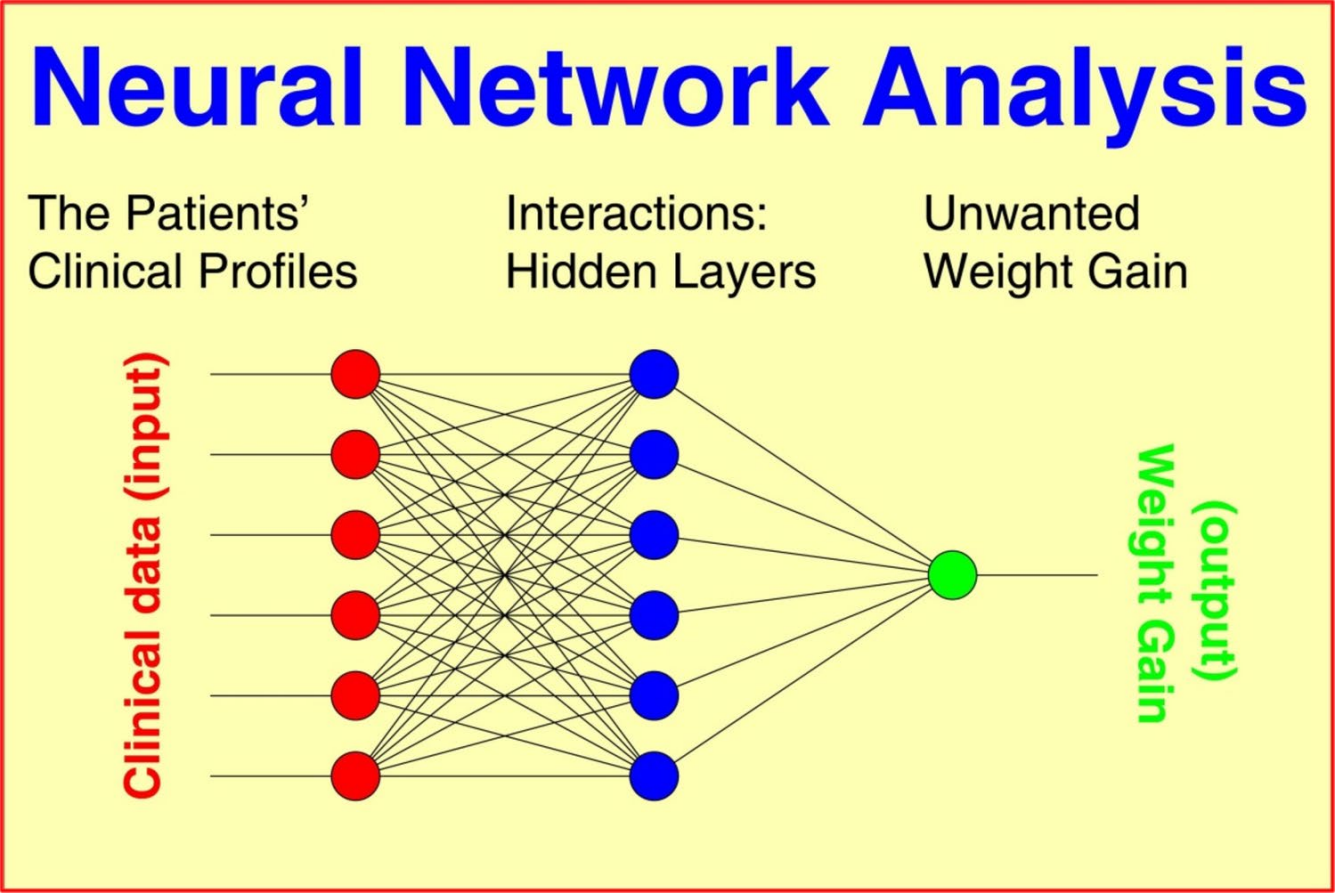
Principal schema of a multilayer Neural Net (NN) where unwanted weight gain (output) results from multiple clinical and nonclinical factors (input) connected to each other by complex interactions via one or more “hidden” layer(s). The NN algorithm iteratively constructs a model that is simultaneously fitted to the observed data of all patients. The achievable goodness of fit depends on the information included, the quality of underlying data, and the number of intermediate layers implemented to model nonlinear interactions

Nonlinear Neural Nets (NN) connect the “neurons” of input and output layers via one or more “hidden” layers (Fig. 1), thus featuring a relatively large number of free parameters. NN connections are realized through (1) weight matrices and (2) model fitting algorithms minimizing an error function in the weight space (goodness of fit). All outputs are computed using sigmoid thresholding of the scalar product of the corresponding weight and input vectors. Outputs at stage “***s***” are connected to each input of stage “***s*** + 1”. The most popular model fitting strategy, the backpropagation algorithm, looks for the minimum of the error function using the method of gradient descent (“steepest descent”). The basic algorithm is:

**Table:** 

(i)	Output:	$s_{i} = \sigma \left[ {\sum\nolimits_{j} {w_{ij} s_{j} } } \right]$	*s*_*i*_*: y*_*i*_ observed	(*i* = *1,2,… N*_i_)
(j)	Hidden layers:	$s_{j} = \sigma \left[ {\sum\nolimits_{k} {w_{jk} s_{k} } } \right]$		(*j* = *1,2,… N*_j_)
(k)	Input:	$s_{k} = x_{k}$	*x*_*k*_ observed	(*k* = *1,2,… N*_k_)
	Improvements:	$\Delta w_{ij} = \alpha \cdot \varepsilon_{i}^{\nu } \cdot s_{j} \cdot s_{i} (1 - s_{i} )$	$\varepsilon_{i}^{\nu } = y_{i}^{\nu } - s_{i}^{\nu }$	(*ν* = *1,2,.. p*)
		$$\Delta w_{jk} = \alpha \cdot \sum\nolimits_{i = 1}^{{N_{i} }} {\varepsilon_{i}^{\nu } } \cdot s_{k} \cdot s_{i} (1 - s_{i} ) \cdot w_{ij} \cdot s_{j} (1 - s_{j} )$$		

where ***x***_k_ denote observed stimuli, ***y***_j_ observed responses, σ the activation function of sigmoid-type: R → (0,1), ***α*** the learning rate, and ***p*** the number of probes (patients). The achievable precision of the model essentially depends on the information included, the quality of underlying data, and the number of intermediate layers implemented to model nonlinear interactions.

Results derived through standard NN approaches, which use 80% of samples for training and the remaining 20% for testing tend to be over-optimistic, in particular in the presence of assessment errors and missing data. By contrast, the ***k***-fold cross-validation approach splits the data into ***k*** roughly equal parts, using ***k***-1 partitions for training, while one partition is used for testing. This process is repeated until each partition has served as a testing set, so that ***k*** estimates of prediction errors are generated. The resulting prediction errors are approximately unbiased for the “true” error for sufficiently large ***k*** (***k*** ≈ 10 is a typical value in practice). In consequence, we relied on the ***k***-fold cross-validation strategy with ***k*** = 10 throughout the entire project and applied the well-proven “random walk” strategy in order to distinguish between local and global minima.

Regarding hyper-parameters, this project relied on the same approach that was successful in our study on inflammatory processes in major psychiatric disorders (70% correct predictions, n = 279 [20]); and in our study on the genetic predisposition to major psychiatric disorders (90% correct predictions, n = 1698 [32]). In detail: (1) to avoid overfitting to a subset of the available data, we used the ***k***-fold cross-validation method described above, which, of course, does not necessarily guarantee good predictions for new, unknown cases; (2) we worked with low learning rates to accurately determine the minimum loss function, as computational load is not a limiting factor for our high-speed servers; (3) accuracy metric was the accuracy score; (4) the learning process stopped prematurely if there was no improvement in 100 cycles; (5) we implemented 1–3 “Hidden Layers” with the number of neurons being systematically varied between 5 and 100 in each layer; and (6) we worked with random weight initialization without pre-training along with sigmoid activation functions.

## Results

### The data material underlying the weight gain analysis

The two patient samples Wave-I (n = 512, 39.6% males) and Wave-II (n = 320, 44.0% males) were carried out using the same study design, the same recruitment procedure, the same assessment instruments, and interviewers trained by the same senior psychiatrists. It can also be assumed that the disorders under study did not change during the observation period [33]. The two samples were therefore quite comparable in their central aspects. By contrast, over the two decades covered by the two samples, a lot has changed: (1) treatment has switched from monotherapy to almost exclusively polypharmacy, as reflected by an increase of concurrently taken drugs from 1.81 ± 1.74 to 4.49 ± 2.5 drugs [*p* < 0.0001; t = 18.24; df = 830]; (2) baseline weight has increased substantially from 72.7 ± 13.7 to 77.5 ± 18.8 kg [*p* = 0.0001; t = 3.91; df = 768]; (3) patients got hospitalized at significantly younger ages: 41.3 ± 12.6 vs. 44.3 ± 13.4 years [*p* = 0.0015; t = 3.189; df = 830]. Most of these changes have not been beneficial to patients.

The combined patient sample included 345 males (ages 40.4 ± 12.9 years) and 487 females (ages 45.2 ± 13.0 years), distributed among the diagnostic groups as follows: F2 diagnoses with 155 males (ages 36.0 ± 12.1 years) and 127 females (ages 39.6 ± 11.4 years); and F3 diagnoses with 190 males (ages 43.9 ± 12.5 years) and 360 females (ages 47.1 ± 13.0 years). The diagnostic groups did not differ in terms of education. In terms of severity at baseline, the F2 patients included 20 mild cases (7.2%) with a PANSS-G baseline score < 30, 124 moderately ill cases (44.4%) with 30 ≤ PANSS-G baseline score ≤ 45, and 135 severely ill cases (48.4%) with a PANSS-G baseline score > 45. Nearly half of the F2 patients (42.3%) displayed significant depression scores.

The F3 sample consisted of 119 mild cases (22.2%) with a HAM-D17 baseline score < 20, 208 moderately ill cases (38.7%) with a 20 ≤ HAM-D17 baseline score ≤ 24, and 210 severely ill cases (39.1%) with a HAM-D17 baseline score > 24. In terms of the HAM-D21 instrument 37.1% of patients reported “paranoid symptoms”, predominantly delusions and hallucinations, and to a much lesser extent, depersonalization and de-realization.

The F2 and F3 samples differed in a variety of ways in so far as F3 patients were older (46.0 years vs. 37.6 years; *p* < 0.0001 [t = − 9.14; df = 830]), were more often assigned to polypharmacy treatment regimens (56.0% vs. 32.3%; *p* < 0.0001 [χ^2^ = 50.7; df = 1]), received more concurrent psychotropic drugs in a polypharmacy regimen (3.2 drugs vs. 2.1 drugs; *p* < 0.0001 [t = − 6.34; df = 830]), and showed more treatment responders under polypharmacy (33.4% vs. 18.7%; *p* = 0.0070 [χ^2^ = 7.30; df = 1]) (Table 1).

**Table 1:** Descriptive statistics of the patient sample comparing the two ICD-10 diagnoses F2 (schizophrenia; n = 282) versus F3 (major depression; n = 550) by means of *t-test* and *chi-square* statistical tests

	F2 diagnoses (n = 282)	F3 diagnoses (n = 550)	
*832 patients under treatment*			
Age (years)	37.6 ± 11.9	46.0 ± 12.9	*p* < 0.0001
Concurrent drugs	2.11 ± 1.94	3.22 ± 2.29	*p* < 0.0001
Day of improvement	13.6 ± 11.2	13.9 ± 10.3	n.s
Day of response	18.1 ± 12.5	24.7 ± 13.1	*p* < 0.0001
Psychotherapy only	0 (0.0%)	24 (4.4%)	
Monotherapy	191 (67.7%)	218 (39.6%)	*p* < 0.0001
Polypharmacy	91 (32.3%)	308 (56.0%)	*p* < 0.0001
Response monotherapy	74 (38.7%)	97 (44.5%)	n.s
Response polypharmacy	17 (18.7%)	103 (33.4%)	*p* = 0.0070
Global side effect score	39.1 ± 20.7	36.4 ± 21.4	n.s
Weight gain	2.36 ± 2.28	2.40 ± 2.27	n.s

Apart from minor age deviations, there were no gender differences between the two diagnostic groups despite differences in sample size (Tables 2, 3).

**Table 2:** Descriptive statistics of the patient sample with ICD-10 diagnosis F2 (schizophrenia; n = 282) comparing males versus females by means of *t-test* and *chi-square* statistical tests

	Males (n = 155)	Females (n = 127)	
*F2-patients under treatment (n* = *282)*			
Age (years)	36.0 ± 12.1	39.6 ± 11.4	*p* = 0.0108
Concurrent drugs	1.83 ± 1.64	2.46 ± 2.39	*p* = 0.0069
Day of improvement	14.4 ± 11.6	12.5 ± 10.5	n.s
Day of response	19.3 ± 13.1	17.0 ± 11.9	n.s
Psychotherapy only	0 (0.0%)	0 (0.0%)	
Monotherapy	108 (69.7%)	83 (65.4%)	n.s
Polypharmacy	47 (30.3%)	44 (34.7%)	n.s
Response monotherapy	35 (32.4%)	39 (47.0%)	*p* = 0.0403
Response polypharmacy	9 (19.2%)	8 (18.2%)	n.s
Global side effect score	35.4 ± 16.4	42.7 ± 23.9	n.s
Weight gain	2.65 ± 2.72	2.05 ± 1. 74	n.s

**Table 3:** Descriptive statistics of the patient subsample with ICD-10 diagnosis F3 (major depression; n = 550) comparing males versus females by means of *t-test* and *chi-square* statistical tests

	Males (n = 190)	Females (n = 360)	
*F3-patients under treatment (n* = *550)*			
Age (years)	43.9 ± 12.5	47.1 ± 13.0	*p* = 0.0058
Concurrent drugs	3.43 ± 2.64	3.11 ± 2.63	n.s
Day of improvement	13.8 ± 10.4	14.0 ± 10.3	n.s
Day of response	23.7 ± 12.6	25.2 ± 14.8	n.s
Psychotherapy only	9 (4.7%)	15 (4.2%)	
Monotherapy	64 (33.7%)	154 (42.8%)	n.s
Polypharmacy	117 (61.6%)	191 (53.1%)	n.s
Response monotherapy	33 (51.6%)	64 (41.6%)	n.s
Response polypharmacy	32 (27.4%)	71 (37.2%)	n.s
Global side effect score	32.5 ± 18.2	39.1 ± 23.1	*p* = 0.0231
Weight gain	2.72 ± 2.41	2.22 ± 2.24	n.s

### Weight gain

In accordance with the literature, the patients of this sample showed an overall higher body weight at study entry compared to the general population. This was reflected by a significant difference in the BMI (*p* = 0.0140 [χ^2^ = 8.54; df = 2]). Here, the overall BMI prevalence of the adult population in Switzerland (2012–2017) served as a comparison. Since the BMI prevalence in the general population 8–10 years ago was slightly lower than today, it can be assumed that the differences to the elevated values of the patients were at least as large as today.

Among the patients with completed weight change trajectories under treatment (n = 491), less than half displayed an essentially unchanged body weight throughout the 3 week observation period, that is, remained within ± 2 kg of their initial weight (47.7%). Less than 5% of patients lost weight with up to 2 kg during the 3 weeks of treatment. A major weight loss > 2 kg was not observed. The other half of the patients (52.3%) experienced significant weight gains: 39.7% in the range of 2–4.9 kg, 8.6% between 5 and 7.4 kg, and 4.1% of 7.5 kg and more (Table 4).

**Table 4:** Treatment-induced weight gain among 491 patients with complete weight gain records

	All patients			Male patients		Female patients
*Weight gain over 3 weeks of treatment*						
− 2.0 kg ≤ w < + 2.0 kg	234	47.7%	90	43.3%	144	50.9%
+ 2.0 kg ≤ w < + 5.0 kg	195	39.7%	85	40.9%	110	38.9%
+ 5.0 kg ≤ w < + 7.5 kg	42	8.6%	20	9.6%	22	7.8%
w ≥ + 7.5 kg	20	4.1%	13	6.3%	7	2.5%
	491		208		283	

More than half of all patients experienced a highly undesirable weight gain of 2 kg or more within 3 weeks of treatment. The male–female differences did not reach statistical significance

This weight gain was largely independent of the patients’ primary diagnosis (F2 vs. F3 patients: 2.36 ± 2.28 kg vs. 2.40 ± 2.27; *p* = 0.5577 [t = 0.587; df = 489]). Even though male patients generally showed higher weight gains than female patients, the respective differences did not reach statistical significance. Specifically, among the F2 patients we found non-significant male–female weight gain differences of 2.65 ± 2.72 kg vs. 2.05 ± 1.74 kg; (*p* = 0.0727 [t = − 1.805; df = 191]), and among the F3 patients those of 2.72 ± 2.41 kg vs. 2.22 ± 2.24 kg; (*p* = 0.1562 [t = − 1.422; df = 294]). There was no significant age dependence either.

### Polypharmacy

**Fig. 2:**
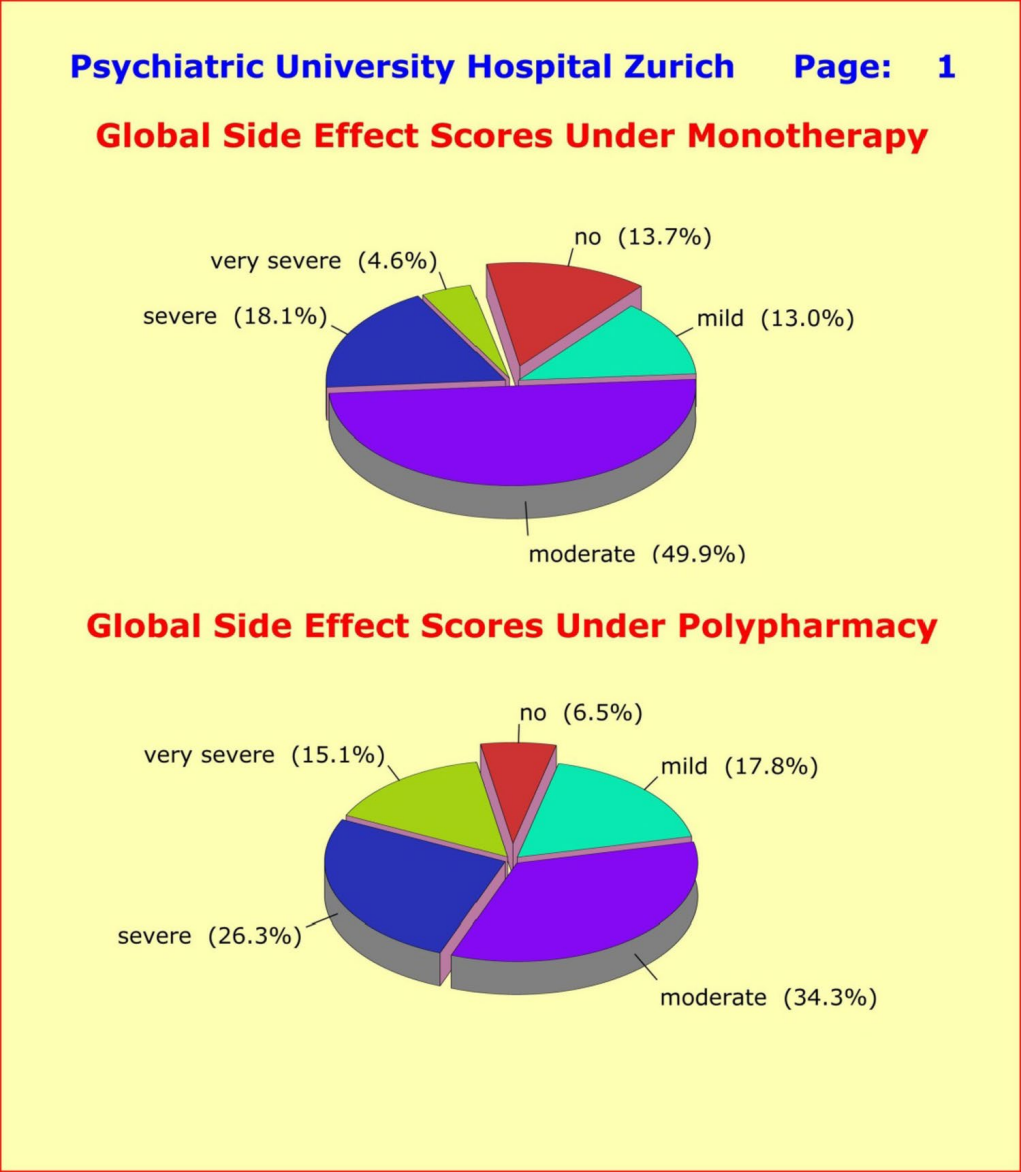
Global side effect score “GS” under monotherapy (upper half: n = 409) versus polypharmacy (lower half: n = 399) assessing «sleep», «appetite», «sexuality», «gastro-intestinal», «cardiac-respiratory», «autonomic», «psychosomatic», «neurological», and «cardiovascular» disturbances. Totally 22.7% of patients under monotherapy reported severe side effects, compared to the significantly higher percentage of 41.4% under polypharmacy. The global side effect score was categorized as follows: GS ≤ 10: no, 10 < GS ≤ 20: mild, 20 < GS ≤ 40: moderate, 40 < GS ≤ 60: severe, and 60 < GS: very severe side effects

The comparison between monotherapy (n = 409) and polypharmacy (n = 399) regimens yielded some unexpected results: (1) far more F3 patients (56.0%) were assigned to a polypharmacy regimen than F2 patients (32.3%) (Table 1); and (2) while the F3 patients did not differ in terms of HAM-D17 severity at entry into the study, the more severe cases among the F2 patients were primarily assigned to monotherapy (PANSS-G baseline scores monotherapy vs. polypharmacy: 49.7 ± 10.9 vs. 35.9 ± 8.8; *p* < 0.0001). Regarding adverse side effects, only a minority reported “no side effects” (8.8%), 17.3% “mild” side effects, 34.9% “moderate” side effects, 25.5% “severe” side effects, and 14.2% “very severe” side effects. In other words, the vast majority of psychiatric patients under psychopharmacological treatment suffered from burdensome and disturbing side effects (Fig. 2).

In line with expectations, patients who received psychotherapy alone without supplementary psychopharmacological treatment experienced only minor side effects (n = 24; global side effect score: 13.6 ± 17.4), followed by patients under monotherapy (n = 409; global side effect score: 27.8 ± 18.5), while the patients under polypharmacy reported more severe side effects (n = 399; global side effect score: 38.5 ± 21.0). The difference between monotherapy and polypharmacy was highly significant (*p* = 0.0011 [t = 3.276; df = 748]). When detailed for gender and primary diagnoses, the respective differences did not reach statistical significance, except for the gender differences among F3 patients (*p* = 0.0231 [t = 2.278; df = 548]) (Table 3).

**Fig. 3:**
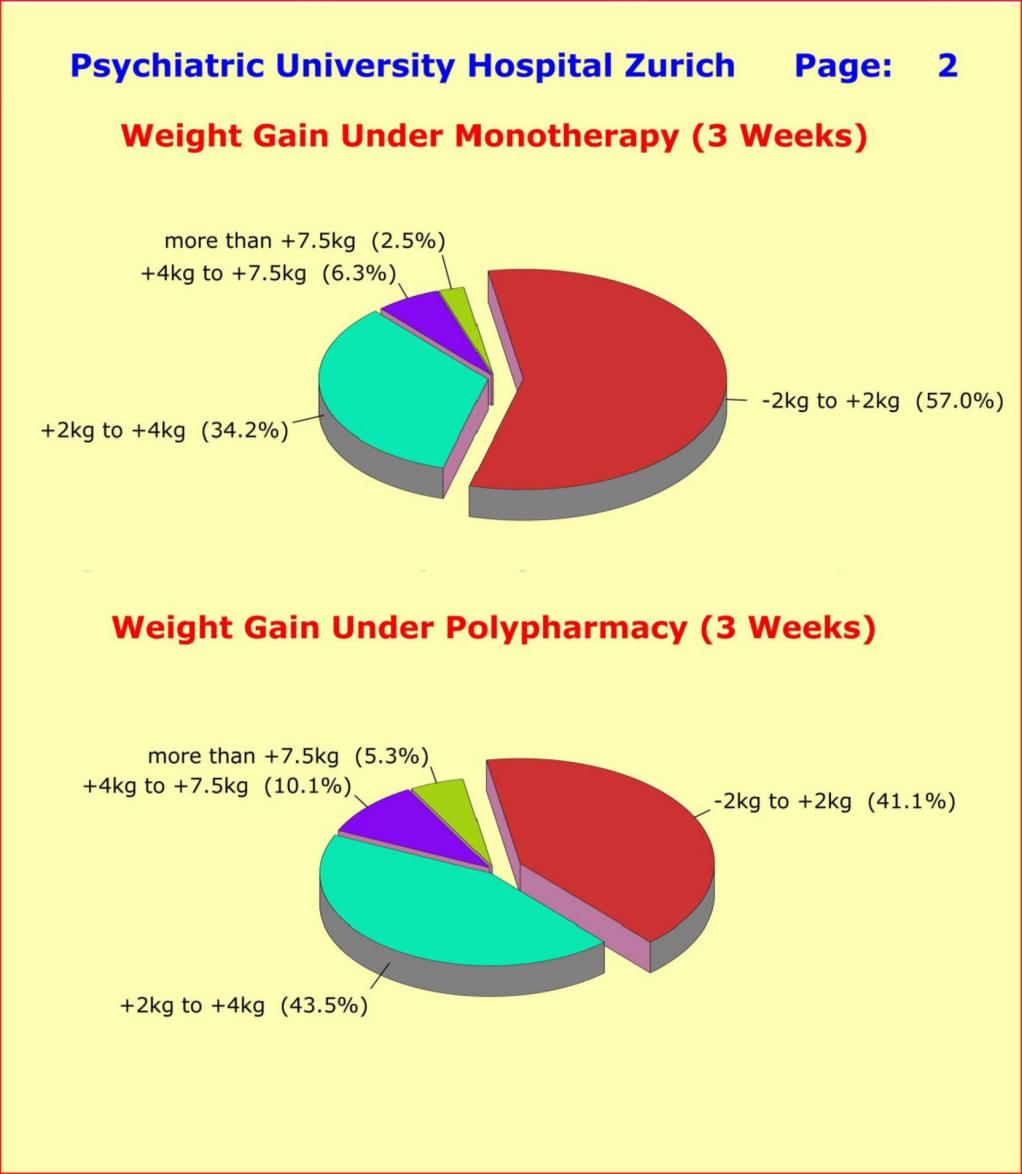
Treatment-induced weight gain among 284 patients under monotherapy (upper half) versus 207 patients under polypharmacy (lower half). Totally 43.0% of patients under monotherapy experienced a highly undesirable weight gain of 2 kg or more within 3 weeks of treatment, while the majority of patients (57.0%) kept their weight “W” virtually unchanged in the range of − 2 kg ≤ W <  + 2 kg. By contrast, totally 59.0% of patients under polypharmacy experienced a highly undesirable weight gain of 2 kg or more within 3 weeks of treatment, compared to only 41.0% who kept their weight “W” virtually unchanged in the range of − 2 kg ≤ W <  + 2 kg

By contrast, the comparison of weight gain between patients under monotherapy versus patients under polypharmacy treatment regimens showed highly significant differences to the disadvantage of polypharmacy patients: 1.98 ± 2.15 kg vs. 2.70 ± 2.32 kg (*p* = 0.0005 [t = 3.505; df = 478]) (Fig. 3).

As to the patients who received psychotherapy only: since repeated measurements of body weight were voluntary, half of these patients (n = 13) had incomplete data regarding unwanted weight gain. Of the remaining patients, 82% showed no weight change. The overall weight gain was 1.48 ± 1.21 kg. It should be noted, however, that this result is only of limited significance due to the small number of cases.

The by far most clinically intriguing finding, though, was that response to treatment was significantly lower under the various polypharmacy treatment regimens compared to monotherapy. That is, among the F2 patients the differences in response rates between monotherapy and polypharmacy were 38.7% vs. 18.7% (*p* = 0.0008 [χ^2^ = 11.2; df = 1]), and among the F3 patients 44.5% vs. 33.4% (*p* = 0.0101 [χ^2^= 6.62; df = 1]). The observed gender differences were marginal (Tables 2, 3).

### Predicting drug-induced weight gain prior to therapeutic intervention

Subsequent correlation analyses revealed several significant interrelations between weight gain and variables that reached significance in an explorative generalized linear regression model (GLM): (1) starting weight (r = 0.11469; *p* = 0.0117); (2) number of concurrent psychotropic drugs (r = 0.16553; *p* = 0.0002); and (3) a treatment-induced “increased appetite” from the very beginning of treatment (r = 0.27525; *p* < 0.0001). No such correlations showed up for the treatment-induced “increased thirst”.

A generalized linear regression model (GLM) combining the above parameters along with gender, age and primary diagnosis explained some 11.5% (r-square = 0.115) of the observed variance in weight gain (dependent variable). A more sophisticated, nonlinear NN model based on Neural Nets could increase the amount of explainable variance somewhat, yet to no more than 30% [variance of correctly classified patients divided by total variance], thus suggesting that unwanted weight gain depended to a larger extent on additional factors, such as personality traits, consumption behavior, lifestyle, and the well-established genetic predisposition to overweight and obesity [34].

Attempts to rank treatment regimens in terms of weight gain, unwanted side effects, time course of improvement, and response to treatment, turned out to be more difficult than expected. Apparently, polypharmacy in clinical reality did not only mean the use of multiple medications concurrently, but it also seemed to involve poly-diversity regarding the combinations of psychotropic drugs used in clinical routine. We counted almost 250 different active compounds amalgamated in numerous combinations, where a mix of antidepressants with antipsychotics seemed to be omnipresent. The sheer number of medications used by the treating psychiatrists of this project produced an enormous diversity of medication combinations. Even though some drug combinations were more common, a clear and widely accepted strategy was not discernible. On average, the patients under polypharmacy treatment regimens received 4.19 ± 1.92 medications, consisting of 3.08 ± 1.81 psychotropic drugs, 0.74 ± 1.15 medications that alleviate adverse side effects and 0.37 ± 0.83 other somatic medications.

In consequence, the vast number of combinations of antidepressants, antipsychotics, and other psychoactive substances observed in this study, —all in widely varying dosages—, made it impossible to reliably rank the various medication regimens in terms of unwanted weight gain on the basis of the available data. Notwithstanding this unsatisfactory result, the strong correlation between unwanted weight gain and the number of concurrent psychotropic medications (r = 0.16553; *p* = 0.0002) clearly called for proper clinical reactions.

### Onset of overweight and interrelations with mental health problems

The BMI data derived from our study of 3180 Students from Europe, the U.S., South America, and China underlined the fact that overweight and obesity have become worldwide problems that often begin to emerge in early life among those affected, independent of any major socio-cultural difference. In fact, overweight and obesity have already reached threatening proportions by the age of 18–22 years. It is an open question whether the observed gender differences will persist over time. For both men and women, the United Kingdom, the U.S., and Argentina lead the ranking, closely followed by the European countries of Italy, Spain, and Switzerland. Students from China make up the rear of the field, only slightly behind (Table 5).

**Table 5:** Overweight and obesity among 3180 university students aged 18–22 years as derived from 8 socio-culturally different sites in Europe, the U.S., South America, and China

University	Sample size	Overweight	Overweight	Obesity	Obesity
		Males (%)	Females (%)	Males (%)	Females (%)
*Overweight & obesity among 3180 students*					
United Kingdom	n = 210 (64 m,146 f)	30.2	27.6	3.2	6.2
Italy	n = 420 (212 m,208 f)	19.1	8.0	1.4	0.0
Spain	n = 400 (202 m,198 f)	20.4	8.2	2.5	2.1
Switzerland (French)	n = 405 (130 m,275 f)	20.9	8.8	0.0	0.0
Switzerland (German)	n = 406 (221 m,185 f)	23.5	9.7	2.3	1.6
USA	n = 407 (180 m,227 f)	33.9	22.7	13.1	9.2
Argentina	n = 500 (138 m,362 f)	32.3	20.7	1.5	5.0
China	n = 432 (222 m, 210 f)	17.1	2.4	2.3	0.0

The data showed that overweight and obesity have become worldwide problems that begin to emerge in early life, largely independent of socio-cultural differences

Using the two socio-culturally independent personality dimensions “activity” and “defeatism” of the COPE instrument in combination with the 5 scales “regular exercises”, “consumption behavior”, “impaired physical health”, “psychosomatic disturbances”, and “impaired mental health” of the Zurich Health Questionnaire ZHQ, we looked for potential interrelations between body weight, personality traits, psychosomatic disturbances, mental health, and physical health.

Correlation analyses revealed a significant interdependence between body weight on the one hand, and the personality traits “activity” (r = − 0.1314; *p* < 0.0001 [− 0.167, − 0.096]) and “defeatism” (r = + 0.0676; *p* = 0.0002 [0.033, 0.104]) on the other. The close link between body weight and alcohol consumption is particularly disturbing (r = + 0.2474; *p* < 0001 [0.216, 0.282]). As to the effects of overweight and obesity on mental health, the empirical data showed that overweight indeed co-occurred with psychosomatic disturbances (r = + 0.1430; *p* < 0.0001 [0.097, 0.166]) and mental health problems (r = + 0.0669; *p* = 0.0003 [0.031, 0.103]) (Table 6). Of course, the observed interrelations must not necessarily be causal. Furthermore, it is worth noting that Table 6 also demonstrates the common truth that regular physical activity has a positive effect on health and body weight.

**Table 6:** Complex interrelations between body weight, the personality traits «Activity» and «Defeatism», consumption behavior, psychosomatic disturbances, and risk of somatic and mental illnesses, estimated through 3180 university students from 8 socio-culturally different sites in Europe, the U.S., South America, and China

	Personality traits, health factors	r	*p*	95% CI
*Body weight, personality traits & general health*				
Body weight	«Activity»	− 0.1314	*p* < 0.0001	[− 0.167, − 0.096]
	«Defeatism»	+ 0.0676	*p* = 0.0002	[0.033, 0.104]
	Alcohol consumption	+ 0.2474	*p* < 0.0001	[0.216, 0.282]
	Psychosomatic disturbances	+ 0.1430	*p* < 0.0001	[0.097, 0.166]
	Illegal drugs	+ 0.0664	*p* = 0.0003	[0.030, 0.103]
	Risk of mental disorders	+ 0.0669	*p* = 0.0003	[0.031, 0.103]
«Activity»	Alcohol consumption	− 0.1336	*p* < 0.0001	[− 0.172, − 0.102]
	Regular exercises	+ 0.1999	*p* < 0.0001	[0.168, 0.236]
«Defeatism»	Illegal drugs	+ 0.1315	*p* < 0.0001	[0.096, 0.166]
	Psychosomatic disturbances	+ 0.2737	*p* < 0.0001	[0.241, 0.306]
	Risk of somatic illnesses	+ 0.1190	*p* < 0.0001	[0.090, 0.160]
	Risk of mental disorders	+ 0.2031	*p* < 0.0001	[0.167, 0.235]

The observed significances (Pearson/Spearman) were generally < 10^–4^, and thus easily survived the very conservative Bonferroni correction for some 30 correlation calculations

The highly significant correlations between the personality trait “defeatism” and (1) psychosomatic disturbances (r = + 2737; *p* < 0.0001 [0.241, 0.306]); (2) the risk of somatic illnesses (r = + 0.1190; *p* < 0.0001 [0.090, 0.160]); and (3) the risk of developing mental disorders (r = + 0.2031; *p* < 0.0001 [0.167, 0.235]), clearly support the hypothesis that a more defeatist personality is more vulnerable to alcohol consumption, while increasing the risk to develop mental health problems.

The above correlations were equally valid across the subgroups, thus emphasizing that these intrinsic interrelations between personality traits, body weight, psychosomatic disturbances, mental disorders, and general health are to a great extent socio-culturally independent. Consequently, the respective personality traits, in particular insufficient coping behavior under chronic stress, appear to be promising targets for psychotherapeutic interventions. Moreover, the COPE instrument that specifically assesses the test persons’ coping behavior may open a way for the early detection of people with an elevated risk of developing mental disorders and obesity.

## Discussion

A drug-induced weight gain of more than 2 kg within 3 weeks of psychotropic drug treatment is a burdensome and quite disturbing experience for patients. More than half of psychiatric patients are affected by this. Addressing quantitatively the intrinsic properties of drug-induced weight gain, we could demonstrate that the complex interrelations between body weight, personality traits, psychosomatic disturbances, mental disorders, and general health can be successfully disentangled on the basis of empirical data.

Although the available data did not readily lead to a comprehensive, clinically applicable model of unwanted weight gain under today’s polypharmacy-oriented treatment regimens, our results have nevertheless revealed ways that have the potential to successfully counteract such weight gain at early stages of treatment. Although this study merely identified factors that are associated with unwanted weight gain, —while the efficacy of the corresponding interventions has not yet been shown through longitudinal studies—, common sense clearly speaks in favor of such interventions, which are easy to implement and do not involve any additional costs:

First of all, unwanted weight gain is highly correlated with the number of concurrently taken medications. A considerable number of patients do not need to be treated with polypharmacy but often have major disadvantages from that. When switching such patients whenever possible to alternative therapeutic approaches, the average weight gain can be expected to decrease quite significantly.

Secondly, baseline weight is also interrelated with unwanted weight gain. In other words, it is highly likely that already overweight patients become even more overweight through psychotropic drugs. Avoiding the use of polypharmacy (as well as monotherapy drugs with a high risk of unwanted weight gain) in patients with overweight can significantly reduce average weight gain.

Thirdly, patients complaining of an “increased appetite” from the very beginning of treatment can be specifically educated regarding their eating behavior and coping strategies, as well as taught to be moderate when eating so that unwanted weight gain is reduced.

Finally, overweight and obesity often begin early in life among those affected, and are interconnected with personality traits, while increasing the risk of developing psychosomatic disturbances, mental health problems, or somatic illnesses. Our student data cleared the way for applications aiming at the early detection and prevention of overweight and obesity. This kind of applications is exactly what we aim to develop through an upcoming longitudinal study.

The use of multiple medications can in *certain cases* be the appropriate and necessary therapeutic option [13, 14, 17–19, 35]. However, polypharmacy-oriented approaches that combine several antidepressants, antipsychotics, mood stabilizers, anxiolytics, hypnotics, antihistamines, and anticholinergics, along with other somatic treatments, are not the appropriate therapy for *everyone*, as it is common practice today. In fact, even though all our results are of a purely statistical nature and only valid for the sample as a whole, —rather than for the individual case—, all empirical data indicate that the majority of F2 and F3 patients *do not* benefit in any respect from polypharmacy treatment regimens: patients experience (1) more and more severe side effects; (2) a significantly increased probability of an unwanted weight gain in the range of more than 2 kg in 3 weeks; and (3) a significantly reduced probability of therapy response. At this point, it is worth noting that (1) active substances, irrespective of their primary sites of action, act completely nonspecifically; (2) antidepressants do not have a specific anti-depressive effect, but very rapidly trigger the onset of improvement in a subset of patients who would not otherwise improve; and (3) antipsychotics do very rapidly reduce positive symptoms, while only partially alleviating general psychopathology and negative symptoms [30, 31].

Given these results, we think that psychiatry has developed to a significant extent in the wrong direction over the past 15 years. In particular, it seems to be time for psychiatry to reconsider its treatment strategies, which are far too one-sidedly fixated on psychopharmacology and pay far too little attention to alternative options, especially in mild cases. Regular exercises and sports can definitely be an effective therapeutic means for a considerable number of cases [36, 37]. GPs are particularly in demand here.

And most importantly, the polypharmacy approach to treating depressive or schizophrenic patients can in no way, not even rudimentarily, solve the problem that there is no causal therapy in psychiatry. Rather, antidepressants and antipsychotics that differ greatly in their biochemical design and primary site of pharmacological action exhibit virtually the same insufficient, non-causal efficacy [30, 31]. Since there will be no causal treatment in the near future either, the revision of therapeutic approaches in psychiatry should have high priority.

The patients of this study all came from Central Europe and are likely to have comparable ancestries with only a modest variation in biological ethnicity (c.f., [32]). It is quite possible that the interrelations found in this study are equally valid for patients of the Northern and Southern parts of Europe, or for U.S. patients of European descent, but may be different for patients of ethnically different populations, for example, Afro-Americans, South Americans, or Asians. On the other hand, our data from 3180 students from different parts of Europe, the U.S., South America, and China have shown that overweight and obesity have become a very comparable worldwide problem, regardless of ethnic and socio-cultural differences. It therefore appears quite likely that studies addressing the same topic will come to similar results —needless to say, that we welcome all studies that give the necessary attention to unwanted, drug-induced weight gain under psychotropic drug treatment.

With regard to our student data, it should be noted that the observed rates of overweight and obesity could well be underestimated, as people who are overweight tend not to face the weight problem, even if surveys are strictly anonymous. Similar biases may also exist with respect to consumption behavior and mental health problems. However, all the above biases should have little influence on the observed interrelations.

*Caveat* It is quite clear that the observed interrelations and the classifiers derived through NN analyses must not necessarily be causal. It could well be the case that the highly significant co-occurrences and the discriminating power inherent in NN classifiers are caused by higher-level factors such as elevated “general vulnerabilities” to physical and mental health problems. It is therefore important to avoid drawing causal conclusions that may result in misleading biases. However, since the treatment options available today for all major psychiatric disorders are non-causal, clinicians are highly interested in reliable tools that (1) can help as to the early detection of risk cases through risk indicators; and (2) can enable “early” interventions, nota bene prior to the development of clinically relevant symptoms.

## Conclusions

More than half of psychiatric patients under psychotropic drugs suffer from a drug-induced weight gain of more than 2 kg within 3 weeks, which is a burdensome and demoralizing experience. This study identified several major factors that contribute to this highly undesirable side effect of psychotropic drug treatment, thereby suggesting clinically easily realizable ways of avoiding such weight gain. The data of 3180 students aged 18–22 years made it clear that overweight and obesity often begin early in life among those affected, and are interconnected with personality traits, while increasing the risk of developing psychosomatic disturbances, mental health problems, or somatic illnesses. The student data also cleared the way for applications aiming at the early detection and prevention of overweight and obesity.

Specifically, the results of this project suggested that psychiatry has developed to a significant extent in an unfavorable direction over the past 15 years. It seems to be time for psychiatry to reconsider its treatment strategies where polypharmacy-oriented approaches play the central role, without taking into account the drawbacks inherent in these strategies.

### Limitations

This project combined data from an 18-year-old study, in which monotherapy was still a common treatment option, with data from a recent study where patients on monotherapy are rare exceptions. Even though the instruments being used in the two studies were the same, inpatients have changed a lot over the years. Unlike two decades ago, one rarely ever finds F2 or F3 inpatients receiving less than two or more medications —even among cases with mild depression. Contributing to a good deal to this development is the widespread pre-treatment of patients with antidepressants and antipsychotics by the family doctors. As a direct consequence, drug-naïve patients are hardly ever seen anymore, neither among hospitalized patients nor among outpatients. Given this ubiquitous development, the combination of our two studies was the only way to “objectively” demonstrate the advantages and benefits of the currently used polypharmacy-oriented treatment strategies in psychiatry. Despite these limitations, we are convinced that the central findings of this project do have general validity and provide a sound basis for direct translations into everyday clinical practice.

## Footnotes

1 There is a third patient sample (“*Response-Genetics Wave-A*”: 2000–2008, n = 397) from additional hospitals with somewhat different recruitment procedures and without interviewer training by us. The three samples constitute our molecular-genetic study on antidepressant/antipsychotic drug response (n = 1,229) [to be published soon].

2 Sustained: no deterioration beyond 15% of achieved baseline score reduction.
